# Effect of Carbonaceous Components on Tribological Properties of Copper-Free NAO Friction Material

**DOI:** 10.3390/ma13051163

**Published:** 2020-03-05

**Authors:** Hsun-Yu Lin, Huy-Zu Cheng, Kuo-Jung Lee, Chih-Feng Wang, Yi-Chen Liu, Yu-Wei Wang

**Affiliations:** 1 Department of Materials Science and Engineering, I-SHOU University, Kaohsiung 84001, Taiwan; carbonfish028@gmail.com (H.-Y.L.); huyzu@isu.edu.tw (H.-Z.C.); 0818yuchen@gmail.com (Y.-C.L.); a0874968@gmail.com (Y.-W.W.); 2 Graduate Institute of Applied Science and Technology, National Taiwan University of Science and Technology, Taipei 10607, Taiwan; cfwang@mail.ntust.edu.tw

**Keywords:** carbonaceous components, non-asbestos organic (NAO) friction materials, coefficient of friction (COF), wear

## Abstract

Copper helps to accelerate heat transfer during the braking process, allowing the brake materials to produce a stable coefficient of friction (COF), which in turn reduces wear loss and braking noise. However, its properties are also quite harmful to aquatic organisms. Finding a suitable replacement that fits all functions of copper for brake materials is not an easy feat. In this paper, six different carbonaceous components (coke, carbon black, carbon fiber, artificial graphite, natural graphite and expanded graphite) were substituted for copper in non-asbestos organic (NAO) friction materials. The hardness, thermal conductivity and tribological behaviors of these copper-free NAO friction materials were examined. Experimental results indicate that carbonaceous components improve lubrication and assist the friction composites with generating friction layers on the worn surface. Specimens containing coke, carbon black or carbon fiber exhibit broken friction layers, whereas specimens containing artificial graphite, natural graphite or expanded graphite exhibit quite adherent and smooth friction layers. Among all the copper-free carbon containing specimens, the specimen containing expanded graphite appears to be the best choice. It has the highest thermal conductivity, a relatively low wear loss and a relatively high and stable COF.

## 1. Introduction

Non-asbestos organic (NAO) friction materials are vital in automotive brake components such as brake shoes, brake pads, brake linings, etc. NAO friction materials composed of fibrous reinforcement, internal lubricant, and friction modifiers are usually bound by phenolic resins [1,2,3]. Copper (Cu) is one of friction materials widely used in brake materials due to its excellent ductility, high conductivity, and good mechanical properties. Cu induces smooth sliding conditions to produce a stable coefficient of friction. Consequently, both the wear loss and braking noise can be largely reduced. Cu also speeds up the thermal energy release during the braking process and reduces the contact temperatures of friction materials, thus avoiding thermal fade.

At present, most of the brake materials contain 1–15% Cu [4,5]. However, as the concentration of copper in water or food exceeds 20 micrograms per gram (µg/g), it can be toxic, especially for aquatic organisms such as to algae, fungi, mollusks and fish. Aquatic organisms are 10 to 1000 times more sensitive to the toxic effects of copper than mammals [6,7,8]. Cu may cause an imbalance in the aquatic food chain and cause rapid death/growth of aquatic organisms. According to earlier research, copper is a neurobehavioral toxicant in fish. In addition, it inhibits olfaction of fish, such as salmon [5,9,10]. Wear debris containing copper may therefore inevitably endanger the marine ecology. For the sustainability of marine ecology, the states of Washington [11] and California [12] both passed legislation limiting the copper content in brake materials in 2010. The development of copper-free friction materials in brake pads thus responds to the above demands. A lot of research or studies on possible ingredients for replacing copper in copper-free NAO friction materials such as organic fibers [13,14,15,16], non-copper metals [17,18,19], solid lubricants [20,21,22,23] and others [24,25,26] have been reported since then.

The solid lubricant, one key component in friction materials, is critically important for the safe and smooth operation of tribological systems. Graphite and other carbonaceous components are well-known nontoxic solid lubricants. These carbonaceous components possess self-lubricating properties, are able to stabilize the coefficient of friction, reduce wear loss and reduce braking noise during the braking process [20,21,22,23,27,28,29,30,31,32,33,34]. Additionally, the high thermal conductivity of carbonaceous components can also accelerate the escaping of the friction heat [20,21,22,23]. With properties similar to those of copper, carbonaceous components may be a suitable replacement as raw materials for copper-free NAO. However, carbonaceous components with varying crystalline structures, crystallinity, size, shape, and appearance may affect the mechanical and thermal properties of friction materials.

Some studies on the tribological performance of various graphite and other carbonaceous components in friction materials are reported [20,21,22,23,27,28,29,30,31,32,33,34]. Kolluri et al. [27] compared the effect of both natural and synthetic graphite and their sizes on fade and recovery properties in friction materials. Natural graphite was found to be more effective to boost friction than synthetic graphite. The fade properties of friction materials were unaffected by the particle size of natural graphite, but fade resistance tended to increase with a decrease in the particle size of synthetic graphite. Cho et al. [28,29] reported the complementary effects between graphite and other solid lubricants in friction materials. Gilardi et al. [20] studied the effects of graphite type on noise reduction in brake pads. They discovered that adding thermal graphite into friction materials resulted in higher thermal conductivity and the better noise reduction performance than when synthetic graphite was added. Aranganathan et al. [21,22,30] concluded that adding thermal graphite in friction materials showed good thermal properties, fade performance, wear resistance, and lubricity in their study and recommended that thermal graphite should be the replacement component for copper. Manoharan et al. [23] compared three kinds of graphite, vein graphite, flake graphite and expandable graphite, as solid lubricants in brake materials and drew the conclusion that brake materials containing expandable graphite exhibited the best thermal stability with good fade and recovery performance among them. Ertan and Yavuz [31] investigated the role of solid lubricants (graphite, coke, and ZnS) on brake performance. They discovered that graphite has a positive effect on the properties of brake linings. However, brake linings containing a high concentration of coke express an unstable coefficient of friction (COF) relationship with the temperature and number of braking cycles. Ahmadijokani et al. [32] investigated the effect of short carbon fibers on the mechanical properties and tribological behaviors of friction materials and discovered that the COF and specific wear rate of friction materials dropped with the addition of carbon fiber. Carbon fiber also aggravated the fade behavior, with a reduction of the COF with temperature increase. Lee et al. [34] studied the effects of two types of liquid impregnant-derived carbon (coal-tar pitch and phenolic resin) on tribological behaviors in a high energy automotive brake system. Results showed that the lamellar carbon transferred from the pitch impregnant generated a lubricating film but the glassy carbon transferred from the resin impregnant demonstrated difficulty in generating a lubricant film. Thus, friction materials with pitch-derived carbon showed more stable tribological performance than those with resin-derived carbon.

Undoubtedly, graphite and other carbonaceous components play important roles in friction materials. However, it is difficult to differentiate whether the composition of carbon adding or the fabrication processes play the key role. In this study, the authors make a systematic comparison among different carbonaceous components. We chose six different kinds of carbonaceous components—coke, carbon black, carbon fiber, artificial graphite, natural graphite and expanded graphite—to replace copper in NAO friction materials. The mechanical, thermal and tribological properties of these copper-free NAO friction materials also were examined.

## 2. Materials and Methods 

### 2.1. Raw Materials

Table 1 lists the formulas of the fabricated specimens. Each specimen contains 14–15 ingredients, including 27.7 vol % binder, 17.5 vol % fibrous reinforcement, 13.9 vol % lubricant, 35.8 vol % friction modifiers and 5.1 vol % theme ingredient (copper components or copper-replaced (carbonaceous) components). Due to business confidentiality, the detailed content ratio of each component is not described here. In Table 2, the parent ingredients, including binder, fibrous reinforcements, lubricants and friction modifiers, were kept fixed at 94.9 vol %. The control specimen containing copper is designated as NAO. The copper-free specimens are obtained by replacing copper with carbonaceous components. Those copper-replaced (carbonaceous) components which had been used in different copper-free specimens are coke (designated as Coke), carbon black (designated as CB), carbon fiber (designated as CF), artificial graphite (designated as AG), natural graphite (designated as NG) and expanded graphite (designated as EG).

Table 3 lists the specification of carbonaceous components used in this study, including the size, thermal conductivity and the manufacturer. Expandable graphite powders (3772, Anthracite Industries, Sunbury, PA, USA) were treated in a microwave oven in ambient conditions at 700 W for 5–10 seconds, 15 times, to form expanded graphite. The morphologies and microstructures of these carbonaceous components were observed using a Hitachi S3400 scanning electron microscope (SEM) (Hitachi Instruments Inc., Tokyo, Japan). The particle sizes of these components were measured using the Horiba LA-350 laser diffraction particle size analyzer (Horiba Scientific, Kyoto, Japan).

### 2.2. Sample Preparation

The sample preparation includes pretreatment of raw materials, mixture of raw materials, hot pressing and post-curing. At first, the aramid pulps were first shaken for one minute (1725 times) using the mixer (8000M Mixer/Mill, SPEX® SamplePrep, Metuchen, NJ, USA). After that, all ingredients were then added into a V-shaped mixer (SY-RBV, Shang Yuh Machine Corp., Ltd, New Taipei City, Taiwan) to mix for 30 min. The mixture was press-molded to a round disk of 25.4 mm in diameter and 10 mm in thickness at 170 °C under a unidirectional pressure of 150 MPa. Finally, the green specimens were then post-cured in an air furnace for four hours at 160 °C.

### 2.3. Hardness Test

The hardness of each specimen was measured by using a hardness test machine (ATK-600, Akashi Corp., Osaka, Japan) according to ASTM E18 standard. A 12.7 mm spherical steel ball shape indenter was used, to which the applied load was set at 100 kgf (HRS). Each set of specimens was tested in five different positions and the average value of hardness was measured.

### 2.4. Thermal Conductivity

The thermal conductivity of each specimen was measured using a thermal conductivity analyzer (Hot disk TPS 2500S, Techmark Precision Instrument Co., Ltd., Göteborg, Sweden) with the transient plane heat source method (ISO 22007-2). A 3.189 mm radius Kapton type sensor was used and placed between two pieces of specimen. The thermal conductivity was measured using input power 50 mW for two to four seconds.

### 2.5. Friction and Wear Test

**Figure 1:**
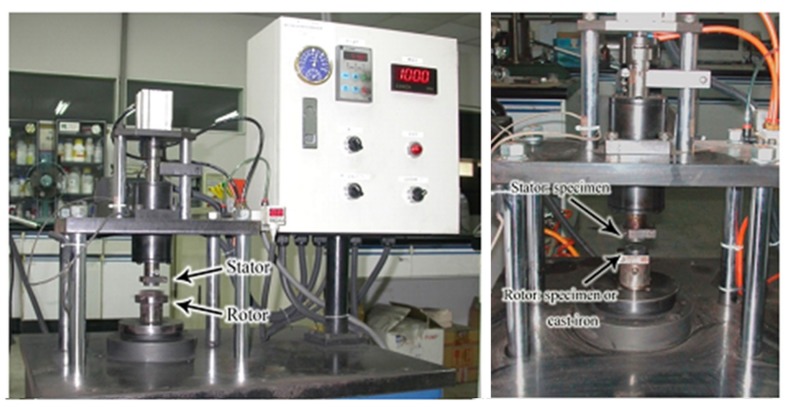
The friction and wear tester.

The friction and wear tests were operated on a homemade disc-on-disc sliding wear tester, as shown in Figure 1. A SAE-G2500 square cast iron (32 mm × 32 mm) was fitted on the rotor as counter face material. Before testing, the specimens and the cast iron were polished using a level of #400 grit paper. A fixed pressure of 1 MPa, constant rotor speed of 800 rpm (linear speed of 0.53 m/s) and testing time of 600 s (sliding distance of 320 m) were used for all wear processes. In order to maintain the similar surface condition of the specimen in each wear test, wear processes were conducted twice as a running-in period before the wear test. After that, wear processes were conducted five times as a wear test for each specimen. At least three specimens were measured for each condition. All tests were performed at room temperature in an atmosphere subject to a relative humidity in the range of 50–70 RH%. The COF was determined from the output of a strain gauge (LRK-100K, NTS Technology, Nara, Japan) and reduced from the formula, $\mu =\frac{M}{r\times F_{n}}$ , where μ is the friction coefficient, M is the moment, F_n_ is the load and r is the radius of specimen. The average COF of each wear test was calculated from the formula $\mu_{\mathrm{avg}}=\frac{\int_{0}^{t}\mu \left(t\right)\mathrm{dt}}{t}$ , where μ_avg_ is the average friction coefficient, μ is the friction coefficient and t is the sliding time. At least 15 wear processes were conducted for each condition, and the average value of the average COF was measured.

The weight difference between before and after five times of wear processes of each specimen was measured with an electronic balance (AG285, Mettler Toledo, Greifensee, Switzerland). As the density is very different from the NAO to the other specimens, the wear weight loss of each specimen was translated into volume loss. The wear volume loss of each specimen was calculated from the formula $V_{\mathrm{loss}}=\frac{W_{\mathrm{loss}}}{D}$ , where V_loss_ is wear volume loss, W_loss_ is wear weight loss and D is density of the specimen. The density of the NAO specimen is 2.28 g/cm^3^, the density of the EG specimen is 1.62 g/cm^3^ and the density of the other specimens is about 1.92–1.94 g/cm^3^. The detailed test method of density is shown in Supplementary Materials (Figure S1).

The morphologies of the polished surfaces and worn surfaces were examined using a SEM (S3400, Hitachi Instruments Inc., Tokyo, Japan). The average surface roughness (R_a_) of the polished surfaces and worn surfaces was measured using a surface profilometer (Surftest SJ-210, Mitutoyo Corp., Kanagawa, Japan). The stylus tip of profilometer was skidded on the surface for 4 mm at a speed of 0.5 mm/s. At least 24 paths were skidded for each condition and the average value of the surface roughness was measured.

## 3. Results and Discussion

### 3.1. Morphologies of Carbonaceous Components

**Figure 2:**
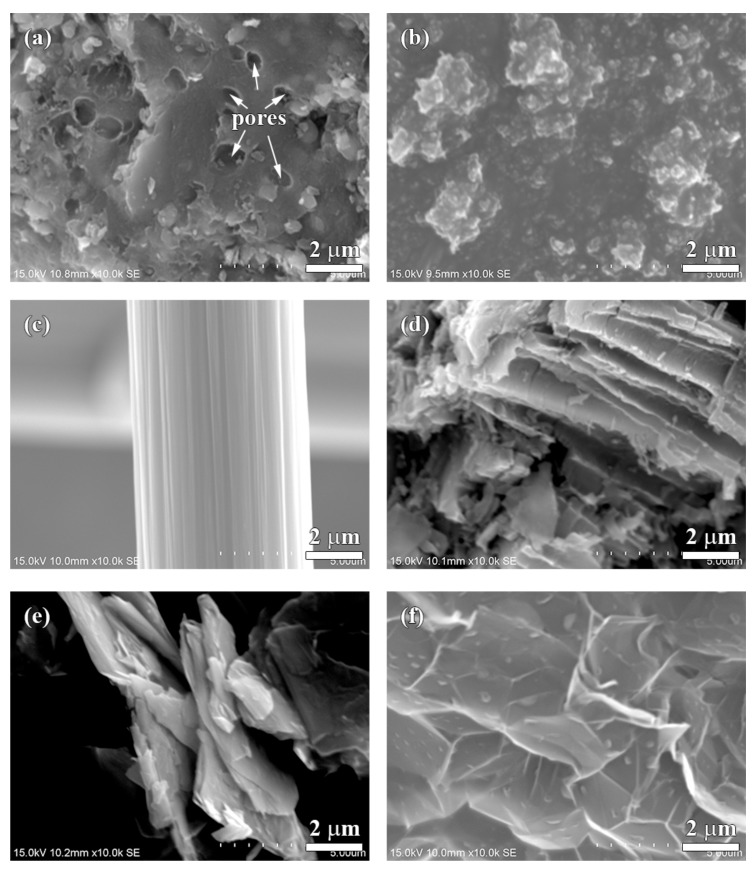
Surface morphologies of different carbonaceous components: (**a**) coke, (**b**) carbon black, (**c**) carbon fiber, (**d**) artificial graphite, (**e**) natural graphite and (**f**) expanded graphite.

The SEM morphologies of the carbonaceous components are shown in Figure 2. Several pores with diameters of less than 1 µm can be observed on the coke particle (Figure 2a). Figure 2b shows an aggregation of carbon black particles whose size is less than 100 nm. The chopped carbon fiber with a clean surface is 7 µm in diameter (Figure 2c). The artificial graphite particles show a layer-like structure (Figure 2d). The natural graphite has a sheet-like and flake shape (Figure 2e). The expanded graphite is composed of several graphite sheets and shows a loose and porous structure (Figure 2f).

### 3.2. Hardness

**Figure 3:**
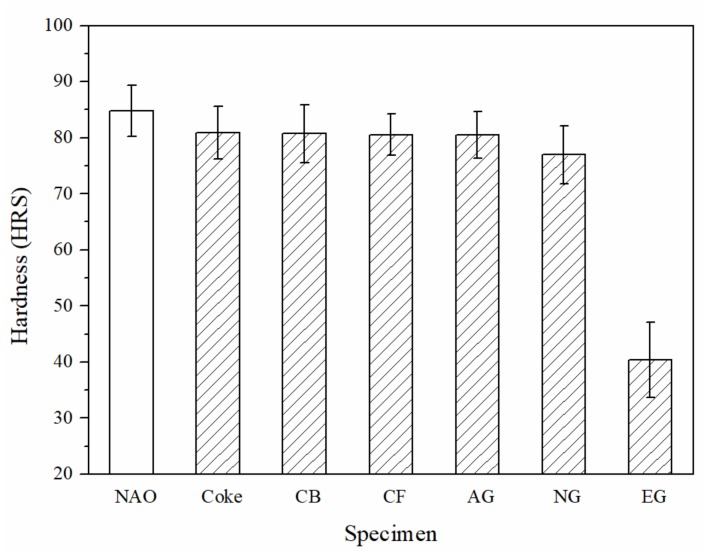
The hardness of specimens.

Figure 3 shows the hardness of each specimen. The hardness of the Coke, CB, CF and AG specimens do not show significant difference (~80 HRS); the hardness of the NG specimen is slightly lower (~77 HRS), but the hardness of EG specimen (~40 HRS) is the lowest among all specimens. The morphology of natural graphite, which easily splits along the slice (Figure 2e), probably explains why the hardness of the NG specimen is slightly lower. For the EG specimen, the morphology of expanded graphite is loose and porous (Figure 2f), resulting in the lowest hardness out of all the specimens.

### 3.3. Thermal Conductivity

**Figure 4:**
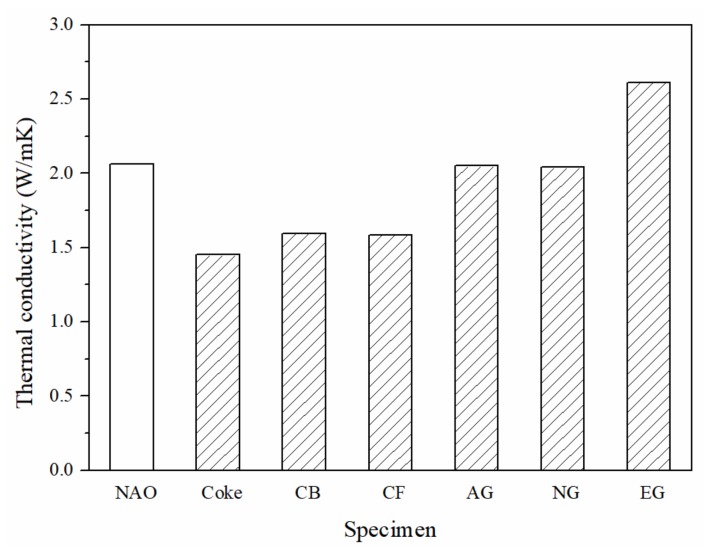
The thermal conductivity of specimens.

Figure 4 shows the thermal conductivity of each specimen. Among the six specimens, the EG specimen shows the highest thermal conductivity (2.6 W/mK). The thermal conductivity of the AG and NG specimens are about the same as the NAO (2.0 W/mK). The CB and CF specimens have lower thermal conductivity (1.6 W/mK) in comparison with the above four, and the Coke specimen has the lowest thermal conductivity of all specimens (1.4 W/mK).

The thermal properties of the carbonaceous components depend on their crystal structure, crystallinity, size, shape, porosity, etc. Carbon atoms in the graphite crystal are in a sp^2^-hybridized state. The theoretical thermal conductivity of graphite along the basal plane can be up to 398 W/mK, but on the vertical basal plane, it is only 2.2 W/mK at room temperature [40]. The expanded graphite is composed of graphite sheets and shows excellent thermal conductivity (a-axis: 400–1300 W/mK, c-axis: 3–65 W/mK) [41]. The thermal conductivity of the Coke, CB and CF specimens is lower than that of the AG and NG specimens, which could be due to the thermal conductivity of coke, carbon black, and carbon fiber (0.2–70 W/mk), which are about one sixth of graphite (a-axis: 398 W/mK, c-axis:2.2 W/mK) [35,36,37,38,39]. Additionally, small pores in the coke, as shown in Figure 2a, result in the Coke specimen having the lowest thermal conductivity among all specimens.

### 3.4. Friction and Wear

**Figure 5:**
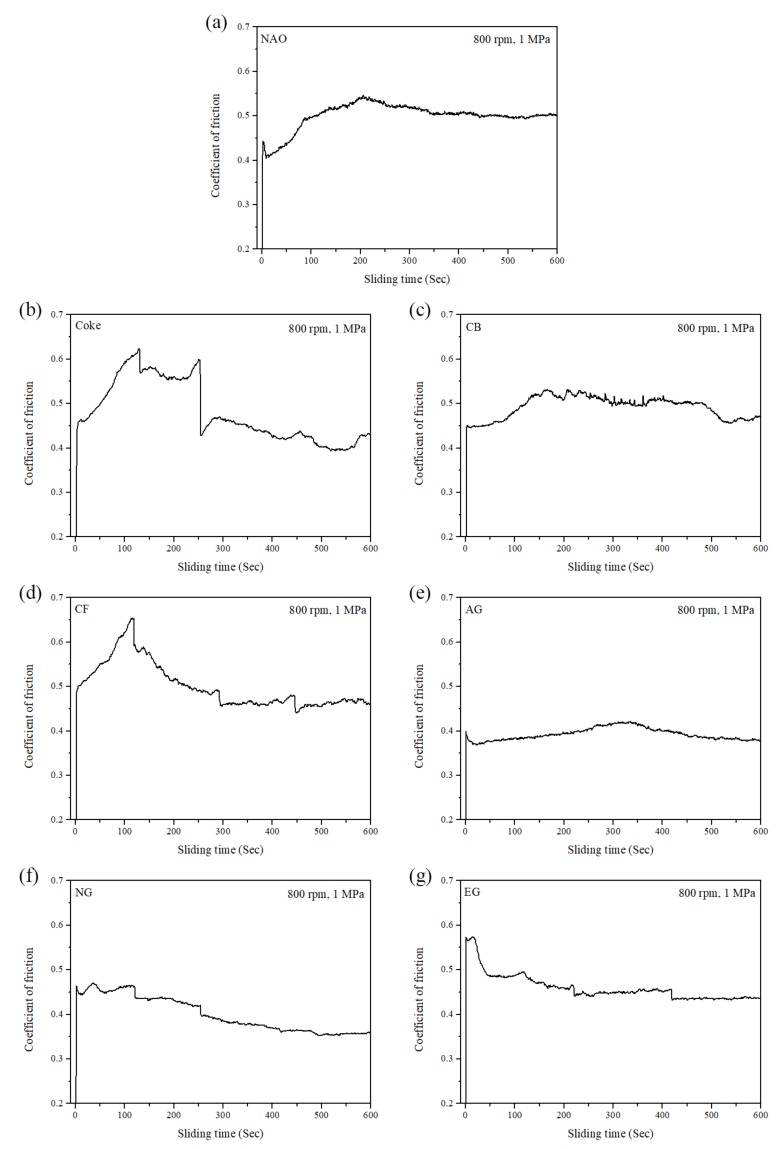
The COF curves of specimens: (**a**) NAO, (**b**) Coke, (**c**) CB, (**d**) CF, (**e**) AG, (**f**) NG, (**g**) EG.

Typical COF curves of specimens under the same pressure (1 MPa) and speed (800 rpm) from at least 15 wear processes are shown in Figure 5. As shown in Figure 5a, the maximum static COF is 0.45, and the kinetic COF is 0.41 at the beginning of the wear test for the NAO specimen. The COF rises up to 0.55 at around 200 s, after which the value keeps at 0.55 from 200 to 600 s.

For the Coke specimen (Figure 5b), the COF rises from 0.46 to 0.63 in the first 130 s and then maintains a high value from 130 to 250 s. However, the COF of the Coke specimen drops down to 0.40–0.45 after 250 s. The COF curve of the Coke specimen is more fluctuant and unstable than that of the other specimens. As shown in Figure 5c, the CB specimen exhibited a similar COF curve as the NAO specimen. Different from the NAO specimen, the CB specimen shows a slight fluctuant curve from 150 to 400 s and exhibits a drop in the COF from 0.50 to 0.45 at around 475 s. In the Figure 5d, the COF of the CF specimen rises from 0.50 to 0.65 at first 115 s and then decreases to 0.47 at around 300 s. After that, the COF curve of the CF specimen becomes stable. The profile of curve is like a stairs shape.

As shown in Figure 5e, the AG specimen showed the most stable COF curve among all the specimens, and the COF is about 0.37–0.40. For the NG and EG specimens (Figure 5f,g), the profiles of the COF curves are also stable; however, the COF curves are like a stairs shape. The EG specimen shows a bit higher COF than the NG specimen.

**Figure 6:**
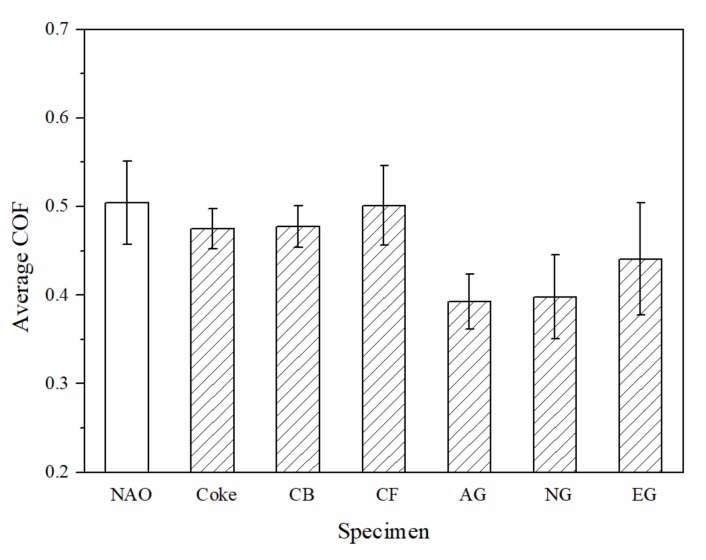
The average COF of specimens.

The average coefficient of friction (COF) of the specimens are shown in Figure 6. Among copper-free carbon containing specimens, the CF specimen shows the highest average COF (0.50), whereas the AG and NG specimens show the lowest average COF (0.39). Except the carbon fiber containing (CF) specimen, the average COF of all the other copper-free carbon containing specimens are lower than the NAO specimen (0.50).

**Figure 7:**
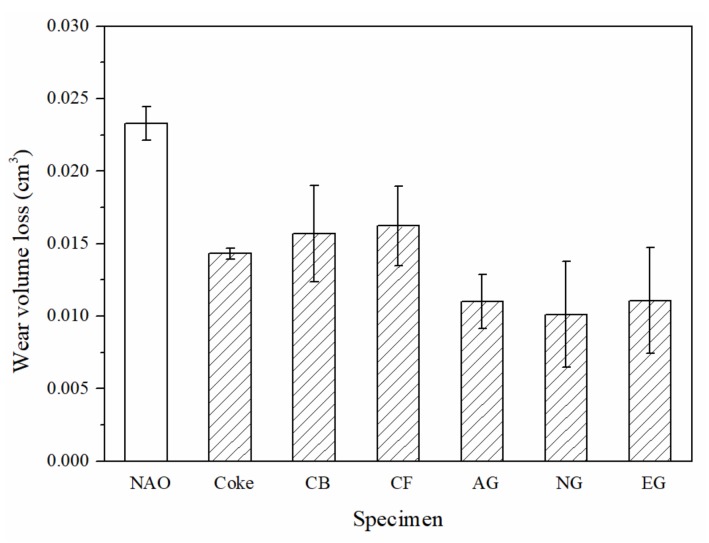
The wear loss of specimens.

Each column of the bar chart in Figure 7 represents the wear volume loss of the specimens. The NAO specimen, containing copper, shows the highest wear loss (0.023 cm^3^) of all the specimens. Among the copper-free carbon containing specimens, the CF specimen shows the highest wear loss (0.016 cm^3^), but the AG, NG and EG specimens show low wear loss (0.010–0.011 cm^3^). Carbonaceous components can improve the wear resistance of the specimens. The wear volume loss of copper-free carbon containing specimens is 30–57% lower than that of the NAO specimen.

In comparison with the NAO specimen, the copper-free carbon containing specimens not only present a lower COF but also lower wear loss, especially the specimens with graphite-like carbonaceous components (i.e., AG, NG and EG). As is commonly known [42,43], graphite is a layered solid with hexagonal lattice. The carbon atoms in the basal plane are bonded with strong covalent bonds. However, between the basal planes is a weak intermolecular force, the Van der Waals force. As a shear force acts on the graphite, the basal planes slide over one another by intracrystalline slip and provide good lubricative effect. The unique structure of graphite is the main reason why graphite-like carbonaceous components (artificial graphite, natural graphite and expanded graphite) show very good lubricating properties.

In addition, coke and the carbon fiber have a high carbon content and a high percentage of non-graphitic structures [44]. Both the quantity and quality of the basal planes of coke or carbon fiber are not as good as graphite, which may result in a higher COF and a higher wear loss of the Coke and CF specimens. On the other hand, the Young’s modulus of carbon fiber is higher than that of other carbon components such as coke and graphite. This higher Young’s modulus, characteristic of carbon fiber, may make the fiber difficult to be compacted. In addition, the stable friction layer on the worn surface is also not easy to form. Therefore, the CF specimen has the highest COF and wear loss in comparison with the other copper-free carbon containing specimens.

Carbon black is composed of several nano scale carbon spheres, which also consist of layers of graphene sheets [44]. However, the lubricity of carbon black is not similar to graphite-like carbonaceous components (artificial graphite, natural graphite and expanded graphite). The CB specimen shows a higher COF and wear than the AG, NG and EG specimens. The size of carbon black (nano-scale) is much smaller than that of graphite-like carbonaceous components (micro-scale). Due to the small size of carbon black, it may be difficult to generate as continuous and stable lubricant film as graphite-like carbonaceous components. The size effect might be the reason for the slight lubricity of the CB specimen.

### 3.5. Morphologies of Polished Surface and Worn Surface

**Figure 8:**
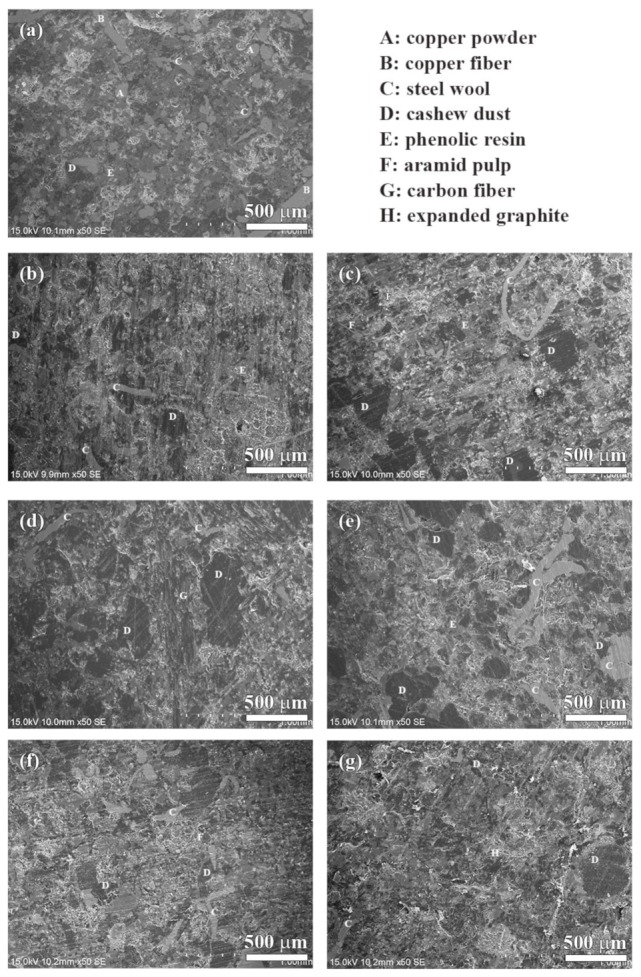
Polished surfaces of specimens: (**a**) NAO, (**b**) Coke, (**c**) CB, (**d**) CF, (**e**) AG, (**f**) NG, (**g**) EG.

The SEM morphologies of the polished specimens are shown in Figure 8. The surfaces were polished using a level of #400 grit paper. As labeled in Figure 8, the different kinds of raw materials were distributed on the polished surface of the specimen, such as copper powder, copper fiber, steel wool, phenolic resin, cashew dust, carbonaceous components and so on.

**Figure 9:**
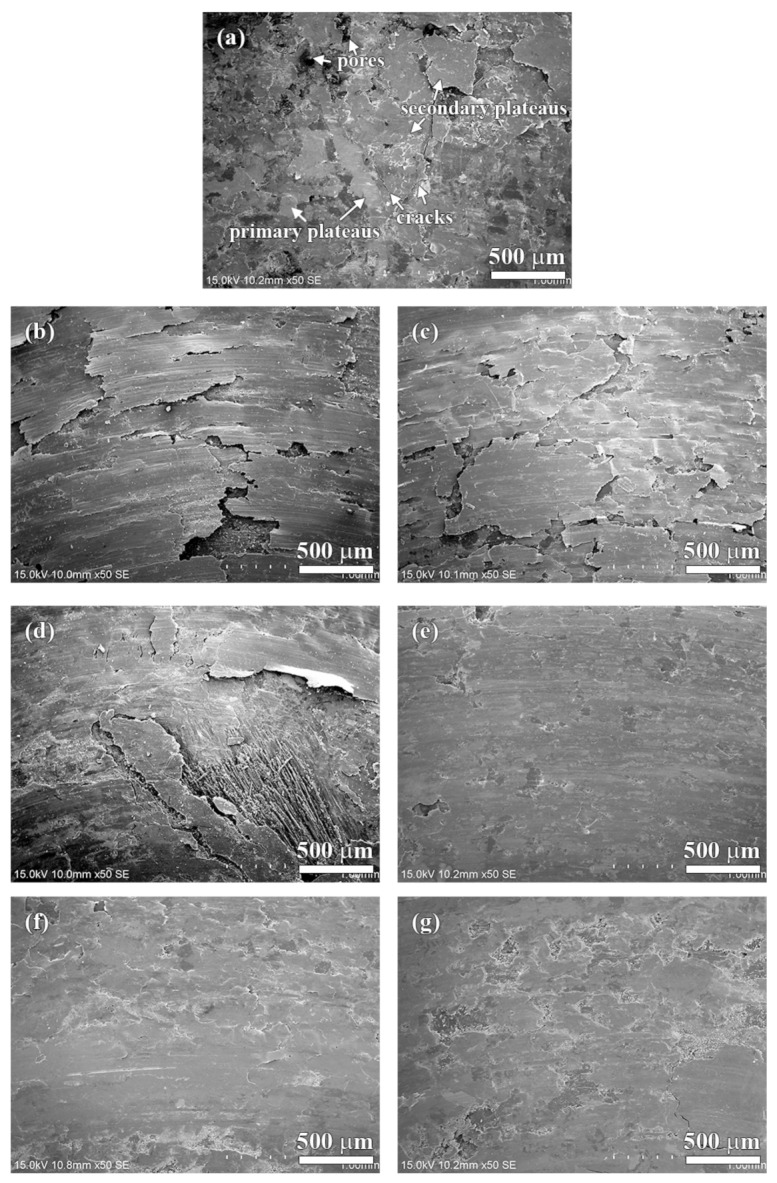
General worn surfaces of specimens: (**a**) NAO, (**b**) Coke, (**c**) CB, (**d**) CF, (**e**) AG, (**f**) NG; (**g**) EG.

**Figure 10:**
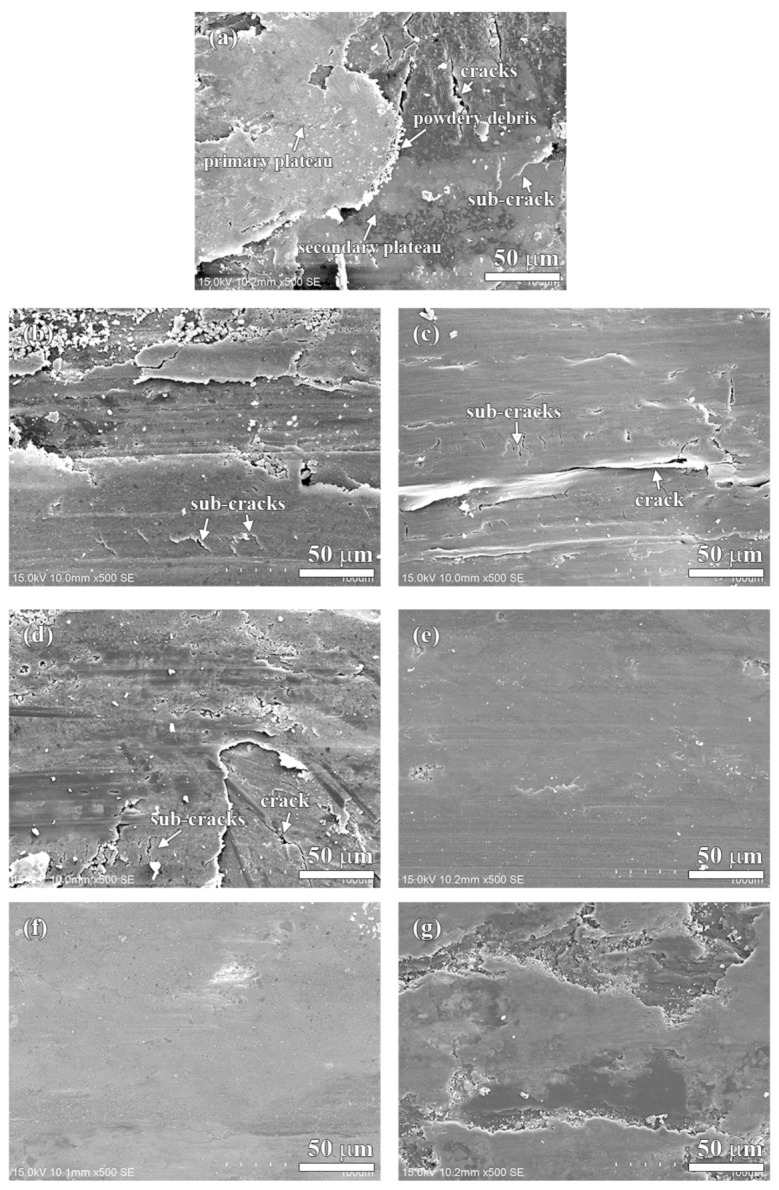
Detailed worn surfaces of specimens: (**a**) NAO, (**b**) Coke, (**c**) CB, (**d**) CF, (**e**) AG, (**f**) NG, (**g**) EG.

The worn surfaces of the specimens are shown in Figure 9 and Figure 10. As shown in Figure 9a, there are several small sized patches (50–200 µm) on the worn surface of the NAO specimen. Some sub-cracks, cracks and pores are observed on the friction layer (as indicated in arrows in Figure 9a and Figure 10a).

In earlier research [45], Eriksson and Jacobson had pointed out two types of contact plateaus, the primary and the secondary plateaus, on the worn surface of organic brake materials. The hard constitutes or fibrous reinforcements with stable mechanical properties and good wear resistance on the worn surface are called primary plateaus. The plateaus which are induced from primary plateaus and made up of compacted/sintered debris are called secondary plateaus. In this study, the primary plateaus (bright patches) and the secondary plateaus (dark patches) were both observed on the worn surface of the NAO specimen (as indicated by arrows in Figure 9a and Figure 10a).

Unlike the NAO specimen, all the copper-free carbon containing specimens exhibited obvious friction layers. Large sized friction layers covered the worn surfaces of the Coke, CB and CF specimens, as shown in Figure 9b–d. However, their friction layers were broken. This might be due to the sub-cracks generated and cracks growing during the wear test, as shown in Figure 10b–d. The broken friction layers may explain why the Coke, CB and CF specimens show higher wear loss than that of the AG, NG and EG specimens. 

The worn surfaces of the AG and NG specimens show large sized, smooth and quite adherent friction layers (Figure 9e,f). There are few cracks and sub-cracks on the friction layers, as shown in Figure 10e,f. The smooth friction layer may be due to the soft and lamellar structure of graphite. In addition, this smooth worn surface also explains the stable COF curve and low wear loss during the wear test.

It should be noticeable that the COF curve of NG is like a stairs shape (Figure 5f), but the curve of AG is not (Figure 5e). However, both the AG and NG specimens show a similar worn surface (Figure 10e,f). This is because the worn surfaces of the specimens are affected by the tribological behaviors of the specimens at the final stage of the wear test. At the final stage of the wear test (400–600 s), the AG and NG specimens exhibited stable COF curves and similar COF values. Therefore, this phenomenon reflects that AG and NG show a similar worn surface. As shown in Figure 9g and Figure 10g, the worn surface of the EG specimen shows few sub-cracks on the large sized, quite adherent friction layer.

### 3.6. Surface Roughness of Polished Surface and Worn Surface

**Figure 11:**
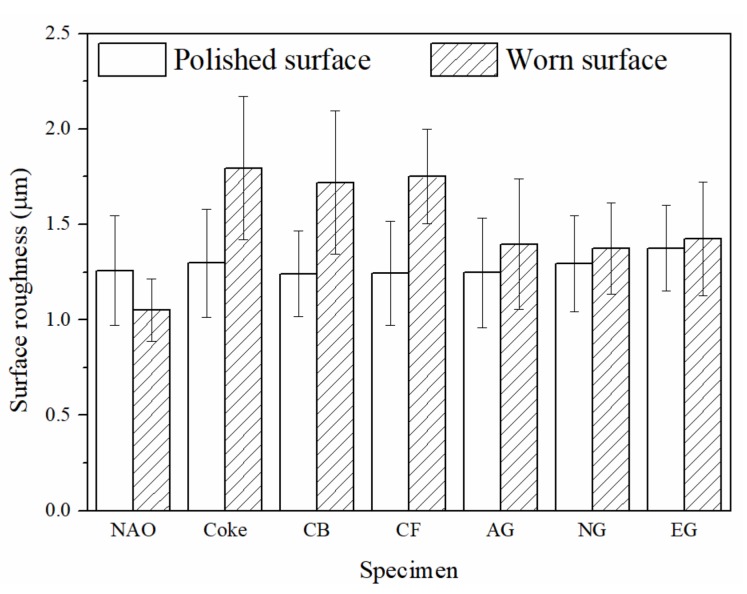
The surface roughness of specimens.

Figure 11 shows the surface roughness of each specimen on the polished surface and worn surface. The polished surfaces were treated with the same condition, therefore the surface roughness of each specimen on the polished surface is close (1.25–1.30 µm), as shown in Figure 11.

After wear test, the surface roughness of the NAO specimen was reduced to 1.05 µm. The results can be explained by soft copper being rubbed to form a smooth friction layer during the wear process. In an earlier study, Kumar and Bijwe [46] also observed a smooth surface topography and appearance of a very thin fine copper enriched friction layer on the worn surface of copper-containing friction materials.

In comparison with the NAO specimen, the copper-free carbon containing specimens show a rough worn surface instead of a polished surface. The friction layers of the copper-free carbon containing specimens are rougher than the copper enriched friction layer of the NAO specimen. For the Coke, CB and CF specimens, the surface roughness of the worn surfaces is about 1.70–1.80 µm. The broken friction layers, as shown in Figure 9b–d, illustrate that the surface roughness of the Coke, CB and CF specimens increases after the wear process. For the copper-free carbon containing specimens, the rougher worn surfaces of the Coke, CB and CF specimens were responsible for the higher COF and larger wear loss during the wear process, as shown in Figure 6 and Figure 7.

The AG and NG specimens appear the smoothest worn surface among all the copper-free carbon containing specimens; the worn surface roughnesses are about 1.38 µm. This is attributed to the fact that the AG and NG specimens show smooth and quite adherent friction layers, as shown in Figure 9e–f. As shown in Figure 9g, the worn surface of the EG specimen is slightly rougher than that of the AG and NG specimens. The worn surface roughness of the EG specimen was 1.42 µm. The smoother worn surface of the AG and NG specimens were also accompanied by a lower COF and smaller wear loss. The EG specimen with a slightly rougher worn surface was accompanied by a higher COF than that of the AG and NG specimens.

The specimens containing graphite-like carbonaceous components show high thermal conductivity, good wear resistance (low wear loss) and a stable and smooth worn surface. In the meantime, the average COFs of the AG and NG specimens 23% lower than that of the NAO specimen. The average COF of the EG specimen is 13% lower than that of the NAO specimen. Among all the specimens, the EG specimen shows the highest thermal conductivity, a relatively low wear loss and a relatively high and stable COF. It seems that expanded graphite has potential to be the candidate to replace copper in copper-free NAO friction materials.

## 4. Conclusions

(1) In comparison with NAO specimens, specimens containing expanded graphite (EG) have a higher thermal conductivity; the specimens containing artificial graphite (AG) or natural graphite (NG) have thermal conductivity similar to the NAO specimen, and the specimens containing coke (Coke), carbon black (CB) or carbon fiber (CF) have lower thermal conductivity.
(2) Most of the carbonaceous components used in this study can improve the tribological performance of specimens, especially graphite-like carbonaceous components, such as artificial graphite, natural graphite and expanded graphite.
(3) The worn specimens containing coke (Coke), carbon black (CB) and carbon fiber (CF) exhibited broken friction layers. The worn specimens containing artificial graphite (AG), natural graphite (NG) and expanded graphite (EG) exhibited quite adherent and large sized friction layers.
(4) After the wear test, the surface roughness of the AG, NG and EG specimens increase slightly. However, the Coke, CB and CF specimens show much rougher worn surfaces.
(5) Among all of the copper-free carbon containing specimens, the specimen containing expanded graphite (EG) has the highest thermal conductivity, a relatively low wear loss and a relatively high and stable COF. It has potential to be the candidate to replace copper in copper-free NAO friction materials.

## Figures and Tables

**Table 1:** Formulation of the friction materials.

Function	Raw Materials	Size (μm)	Content (vol %)
Binder	phenolic resin	45	27.7
Fibrous reinforcement	aramid pulp potassium titanate steel wool	5–20 ^1^ 3–5 ^1^ 10–100 ^1^	17.5
Lubricant	antimony trisulfide artificial graphite	45 75	13.9
Friction modifier	aluminum barite cashew dust nitrile butadiene rubber (NBR) silica silicon carbide zirconia	45 20 20–500 120 45 40 13.8	35.8
Theme ingredient	copper (Cu powder/Cu fiber) or copper-replaced (carbonaceous) components	50 ^2^/ 60–100 ^3^ (see Table 3)^4^	5.1

^1^ Diameter of fibrous reinforcement. ^2^ Size of copper powder. ^3^ Diameter of copper fiber. ^4^ Size of copper-replaced (carbonaceous) components.

**Table 2:** List of copper and copper-replaced (carbonaceous) components (content in vol %).

Ingredient	NAO	Coke	CB	CF	AG	NG	EG
Parent ingredient ^1^	94.90	94.90	94.90	94.90	94.90	94.90	94.90
copper powder	2.55	-	-	-	-	-	-
copper fiber	2.55	-	-	-	-	-	-
coke	-	5.10	-	-	-	-	-
carbon black	-	-	5.10	-	-	-	-
carbon fiber	-	-	-	5.10	-	-	-
artificial graphite	-	-	-	-	5.10	-	-
natural graphite	-	-	-	-	-	5.10	-
expanded graphite	-	-	-	-	-	-	5.10

^1^ Parent ingredient contains binder, fibrous reinforcements, lubricants and friction modifiers.

**Table 3:** Carbonaceous components used in this study.

Carbonaceous Component	Size (μm)	Thermal Conductivity (W/m K)	Description, Company
coke	D50^3^:43.9 D95^4^:129.9	< 0.973 [35]	Metallurgical coke, China Steel Chemical Corp., Kaohsiung, Taiwan
carbon black	D50:0.9 D95:2.4	0.2–0.3 [36,37]	FEF N-550, China Synthetic Rubber Corp., Kaohsiung, Taiwan
PAN^1^-based carbon fiber	diameter:7 length:3000–5000	8–70 [38,39]	T700S, Toray Industries, Inc., Tokyo, Japan
artificial graphite	D50:77.8 D95:204.6	a-axis:398 c-axis:2.2 [40]	G-3, Hsu I Enterprises Corp., Tainan, Taiwan
natural graphite	size:10–20 thickness: <3	a-axis:398 c-axis:2.2 [40]	Graphite flake, -325 mesh, Alfa Aesar, Ward Hill, MA, USA
expanded graphite^2^	size:25 expanded length:25–7500	a-axis:400–1300 c-axis:3–65 [41]	Expandable graphite, 3772, Anthracite Industries, Sunbury, PA, USA (Expansion ratio ~1:300)

^1^ Polyacrylonitrile (PAN). ^2^ The expandable graphite was treated in a microwave to form expanded graphite.^3^ D50: the size below which 50% of powder sample is contained. ^4^ D95: the size below which 95% of powder sample is contained.

## Supplementary Materials

The following are available online at https://www.mdpi.com/1996-1944/13/5/1163/s1. Supplementary materials contain the bulk density of each specimen (Figure S1 Bulk density of specimens).
