# Supplementary material for: Effect of Carbonaceous Components on Tribological Properties of Copper-Free NAO Friction Material
Source: Materials (Basel). 2020 Mar 5;13(5):1163. doi: 10.3390/ma13051163 (PMC7084987; doi:10.3390/ma13051163)
Supplement: Supplementary file 1 [file materials-13-01163-s001.pdf]

materials

Article

Effect of Carbonaceous Components on Tribological Properties of Copper-Free NAO Friction Material

Hsun-Yu Lin [1], Huy-Zu Cheng [1], Kuo-Jung Lee [1,*], Chih-Feng Wang [2], Yi-Chen Liu [1] and Yu-Wei Wang [1]

[1] Department of Materials Science and Engineering, I-SHOU University, Kaohsiung 84001, Taiwan; carbonfish028@gmail.com (H.-Y.L.); huyzu@isu.edu.tw (H.-Z.C.); 0818yuchen@gmail.com (Y.-C.L.); a0874968@gmail.com (Y.-W.W.)

[2] Graduate Institute of Applied Science and Technology, National Taiwan University of Science and Technology, Taipei 10607, Taiwan; cfwang@mail.ntust.edu.tw

* Correspondence: krlee@isu.edu.tw; Tel.: +886-7-6577711 (3126)

Received: 31 December 2019; Accepted: 2 March 2020; Published: date

Bulk density

The bulk density of each specimen was measured using porous solid density tester (TWS-300PY, MatsuHaKu, Taiwan) with the water immersion method (ASTM-C20) and calculated from the following equation:

$$B = \frac{D}{W - S} \quad (1)$$

where *B* is bulk density, *D* is dry weight, *S* is suspended weight, and *W* is the saturated weight.

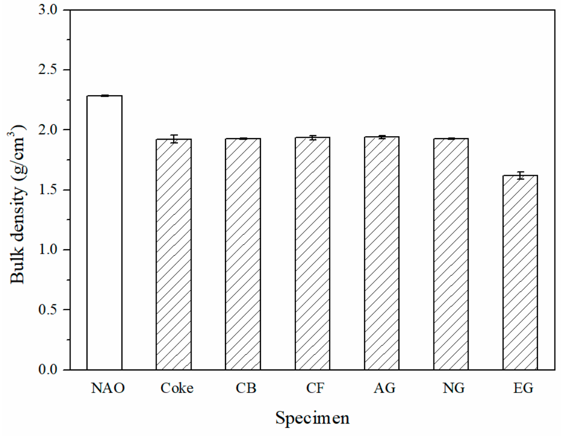

Figure 1. Bulk density of specimens.

Figure S1 shows the bulk density of each specimen. The density of copper components is about two to three times higher than that of carbonaceous components. NAO specimen shows the highest density (2.28 g/cm^3) of all specimens. With the exception of the expanded graphite-containing specimen, all other carbon- containing specimens show the close bulk density (1.92–1.94 g/cm^3). Due to a loose and porous structure of expanded graphite, EG specimen shows the lowest bulk density of all specimens (1.62 g/cm^3).

© 2020 by the authors. Submitted for possible open access publication under the terms and conditions of the Creative Commons Attribution (CC BY) license (http://creativecommons.org/licenses/by/4.0/).
